# Paramedic use of the Physician Order for Life-Sustaining Treatment (POLST) for medical intervention and transportation decisions

**DOI:** 10.1186/s12873-022-00697-3

**Published:** 2022-08-11

**Authors:** Amelia M. Breyre, Karl A. Sporer, Glen Davenport, Eric Isaacs, Nicolaus W. Glomb

**Affiliations:** 1 University of California San Francisco, Department of Emergency Medicine, San Francisco, USA; 2 Alameda County Emergency Medical Services Agency, San Leandro, USA; 3 Columbia University, Columbia Center for Teaching and Learning, Oregon, USA

**Keywords:** POLST, Hospice, Palliative care, Serious illness

## Abstract

**Background:**

Physician Order for Life-Sustaining Treatment forms (POLST) exist in some format in all 50 states. The objective of this study is to determine paramedic interpretation and application of the California POLST for medical intervention and transportation decisions.

**Methods:**

This study used a prospective, convenience sample of California Bay Area paramedics who reviewed six fictional scenarios of patients and accompanying mock POLST forms. Based on the clinical case and POLST, paramedics identified medical interventions that were appropriate (i.e. non-invasive positive pressure airway) as well as transportation decisions (i.e. non-transport to the hospital against medical advice). EMS provider confidence in their POLST interpretation was also assessed.

**Results:**

There were 118 paramedic participants with a mean of 13.3 years of EMS experience that completed the survey. Paramedics routinely identified the selected medical intervention on a patients POLST correctly as either *comfort focused*, *selective* or *full treatment* (113-118;96%-100%). For many clinical scenarios, particularly when a patient’s POLST indicated *comfort focused treatment*, paramedics chose to use online medical oversight through base physician contact (68-73;58%-62%). In one case, a POLST indicated “*transport to hospital only if comfort needs cannot be met in current location*”, 13 (14%) paramedics elected to transport the patient anyway and 51 (43%) chose “Non-transport, Against Medical Advice”. The majority of paramedics agreed or strongly agreed that they knew how to use a POLST to decide which medical interventions to provide (106;90%) and how to transport a patient (74;67%). However, after completing the cases, similar proportions of paramedics agreed (42;36%), disagreed (43;36%) or were neutral (30;25%) when asked if they find the POLST confusing.

**Conclusion:**

The POLST is a powerful tool for paramedics when caring patients with serious illness. Although paramedics are confident in their ability to use a POLST to decide appropriate medical interventions, many still find the POLST confusing particularly when making transportation decisions. Some paramedics rely on online medical oversight to provide guidance in challenging situations. Authors recommend further research of EMS POLST utilization and goal concordant care, dedicated paramedic POLST education, specific EMS hospice and palliative care protocols and better nomenclature for non-transport in order to improve care for patients with serious illness.

**Supplementary Information:**

The online version contains supplementary material available at 10.1186/s12873-022-00697-3.

## Background

The Physician Orders for Life-Sustaining Treatment (POLST) forms are signed medical orders, voluntarily completed by patients with serious illness to communicate their preferences for medical care. It was first designed in 1994 by ethicists in Oregon, but now exist in some form in all 50 states and is a common adjunct to Advanced Directives [1–3]. By design, the POLST has three intended audiences: patients, physicians, and Emergency Medical Services (EMS). The POLST is a medical order designed for patients in cardiopulmonary arrest (section A) and for patients with a pulse and/or breathing (section B) and honored by EMS. Each section of the POLST provides bundled options with common treatments appropriate for or excluded at the different levels of care, medical interventions such as intubation, cardioversion, intravenous fluid hydration and transportation [2].

Despite their ubiquity, there are a very limited number of studies about EMS provider use, interpretation and application of POLST forms both in and out of cardiopulmonary arrest [4–8]. One survey of Emergency Medical Technicians concluded that 93% found the forms useful in cardiopulmonary arrest but only 63% found the form helpful in guiding treatment for patients with a pulse and breathing [4]. In a separate study by Mirarchi et al., significant variation in EMS provider response to POLST forms and case scenarios demonstrated underlying confusion in understanding the Pennsylvania POLST [6]. One study of out-of-hospital cardiac arrest in Oregon found out-of-hospital care and emergency department care generally concordant to POLST documentation [7]. A systematic literature review found moderate strength of evidence that treatment limitations documented on POLSTs, may reduce treatment intensity for patients with serious illness in the prehospital setting, however limitations in analysis did not allow an understanding of specifically when discordant interpretation occurs [8].

**Fig. 1:**
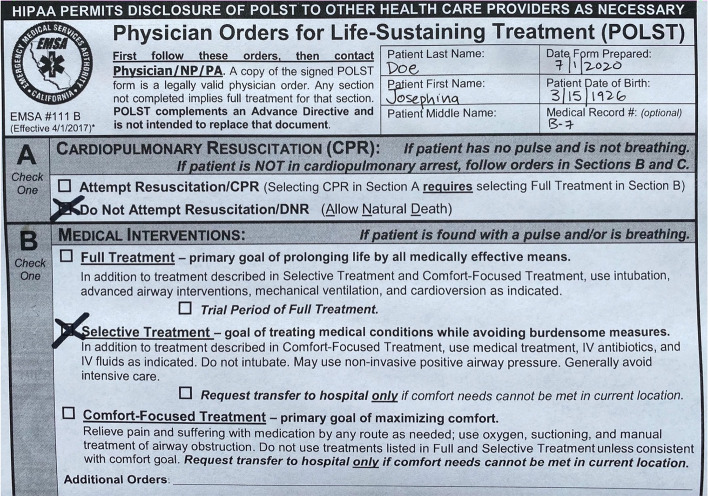
Mock California POLST

Many variations of the POLST, including California, includes a medical order that explicitly states “*transfer to hospital if treatment needs cannot be met in current locations*” (Fig. 1). This medical order is clearly intended specifically for EMS providers. However, how this might be interpreted and applied in clinical scenarios is unknown. The EMS system was historically designed for swift resuscitation and transportation of patients, the decision for non-transport of patients with serious illness may present a challenging scenario.

### Objective

The objective of this study is to determine paramedic interpretation and application of the California POLST for medical intervention and transportation decisions for patients not in cardiopulmonary arrest.

## Methods

### Study design

This was a prospective study, approved by the University of California, San Francisco Institutional Review Board, that used an internet-based survey (Google forms, Mountainview, CA & Qualtrics, Seattle, WA) with a convenience sample of California Bay Area EMS providers between September 2021 and November 2021. In the state of California, the POLST is approved by the state EMS Agency, however EMS regulation, protocol development and education are mostly the responsibility of the local EMS Agency (LEMSA) level. LEMSA and stakeholders (Alameda, Contra Costa, San Francisco, Coastal Valleys, San Mateo) were asked to disseminate a recruitment letter from the author, requesting distribution of the survey link to paramedics. Only paramedics were included in this study because all transporting agencies used primarily Advanced Life Support units and types of medical interventions in the survey were appropriate to paramedic scope of practice in the region.

Participants were asked to review fictional cases of patients not in cardiopulmonary arrest and accompanying fictional mock California POLST forms (Fig. 1, Table 2). On the California POLST, for patients in cardiopulmonary arrest there is an option to “*Attempt Resuscitation*” or “*Do Not Attempt Resuscitation/DNR”* (POLST Part A)*.* For patients with a pulse or breathing there is an option for “*Full Treatment*”, “*Selective Treatment*” and “*Comfort Focused Treatment*” (POLST Part B). A panel of local California Emergency Medicine, EMS and palliative care experts reviewed cases for appropriateness and determined correct answers (in green, Table 2). Scenario 2 and Scenario 6 are identical clinical cases used to assess internal consistency of participants.

Prior to the cases, participants were asked questions regarding participant confidence using the POLST for medical interventions and transportation decisions with a Likert scale. After the cases, participants were asked if they found the POLST “confusing” and about their experience with POLST training (Supplement 1). The cases were piloted on four paramedics prior to distribution. They reviewed the pilot survey and provided input on readability, clinical appropriateness, and length of time for completion. Feedback was integrated into the survey design.

Responses were anonymous. The total survey was approximately 20 min in length and participants received $20 gift card for participation. The Strengthening the Reporting of Observational Studies in Epidemiology (STROBE) guidelines were applied [9].

### Analysis

For each of the six cases, participant responses were evaluated for minimum correct response based on expert panel consensus as described above. An analysis of variance (ANOVA) using SPSS (IBM SPSS 25, Armonk, NY) was used to evaluate statistical difference between experience in EMS and participant self-assessed Likert scale of confidence and confusion using the POLST.

## Results

### Characteristics of study subjects

There were 118 study participants from six Bay Area counties in California. The mean age of participants was 39.5 years (median 36.0, IQR 30.0–43.3, range 23–60) with a mean of 13.3 years of EMS experience (median 11.0, IQR 5.1–16.9; range 2–38 years;). The majority of participants identified as male (92;78%) and were fire-based EMS (66;56%). Only 47 (40%) of participants reported prior formal training on POLSTs. The summary of participant demographic data is in Table 1.

**Table 1:** Demographic description of survey participants

Characteristic	Median	Minimum	Maximum
Age in years	36.0 (IQR: 30.0–43.3)	26	60
# of years experience in EMS	11.0 (IQR: 5.1–16.9)	2	38
Gender	Frequency	% of Total (N = 118)	
Female	26	22%	
Male	92	78%	
Type of EMS System			
Fire-based	66	56%	
Private	52	44%	
Prior training on POLST			
No	24	20%	
Yes – self-training/ on the job	47	40%	
Yes – formal training	47	40%	

### Main Results

The summaries of paramedic identification of treatment interventions and transportation decisions are in Table 2. When a POLST indicated *comfort focused treatment* (Case #2, #6), or *selective treatment* (Case #1, #4, #5), paramedics routinely identified the correct treatment selection (96%-99%). Paramedics unanimously (118;100%) identified patient’s preference for *full treatment* (Case #3).

**Table 2:** EMS provider interpretation of POLST for treatment interventions and transportation

Case and POLST	Paramedic Interpretation and Treatments Selected			
**Case # 1:** A 95-year-old female with dementia lives with her family is more confused than her baseline. She has foul smelling diapers and a chronic pressure sacral ulcer. Vital Signs P, 105; RR 12; SaO2, 97%; T, 39 C; BP, 90/50 **POLST Selection:** DNR/DNAR, Selective Treatment *Correct minimum:* IV normal saline AND transport to the hospital		**Comfort Focused**	**Selective Treatment**	**Full Treatment**
	# of Respondents:	0	**117 (99%)**	1 (1%)
	Advanced Airway		1 (1%)	0
	Bag Valve Mask		43 (36%)	1 (1%)
	NIPPV		36 (31%)	1 (1%)
	Supplemental oxygen		76 (64%)	1 (1%)
	IV normal saline		112 (95%)	0
	IV Fentanyl		49 (42%)	1 (1%)
	Naloxone		23 (19%)	1 (1%)
	Contact Base Physician		27 (23%)	1 (1%)
	Transport the patient to the hospital		112 (95%)	1 (1%)
	Non-transport, Assess & Refer		1 (1%)	0
	Non-transport, Refusal of Service		3 (3)	0
	Non-transport, AMA		5 (4%)	0
**Case #2:** A 43-year-old female with metastatic ovarian cancer on hospice feels very short of breath because of fluid that has accumulated in her lungs. Vital Signs P, 115; RR, 30; SaO2, 86% 2L home oxygen; T, 37 C; BP, 130/70. She states that she wants help with her symptoms, but does not want to go to the hospital **POLST Selection:** DNR/DNAR, Comfort Focused Treatment *Correct minimum:* Non-transport		**Comfort Focused**	**Selective Treatment**	**Full Treatment**
	# of Respondents:	**113 (96%)**	2 (2%)	3 (3%)
	Advanced Airway	1 (1%)	0	0
	Bag Valve Mask	14 (12%)	0	0
	NIPPV	29 (25%)	1 (1%)	1 (1%)
	Supplemental oxygen	101 (86%)	1 (1%)	3 (3%)
	IV normal saline	22 (19%)	1	0
	IV Fentanyl	39 (33%)	0	0
	Naloxone	7 (6%)	0	0
	Contact Base Physician	73 (62%)	1 (1%)	1 (1%)
	Transport the patient to the hospital	12 (10%)	1 (1%)	0
	Non-transport, Assess & Refer	22 (19%)	0	0
	Non-transport, Refusal of Service	19 (16%)	0	0
	Non-transport, AMA	68 (58%)	1 (1%)	2 (2%)
**Case #3:** A 87-year-old male with advanced dementia, on apixaban for atrial fibrillation hits his head at his nursing facility and now has a scalp hematoma. He is awake and talking and to staff at his mental status baseline. Vital Signs P, 75; RR, 12; SaO2, 99%; T, 37 C; BP, 115/75 **POLST Selection:** DNR/DNAR, Full Treatment *Correct minimum:* Transport to the hospital		**Comfort Focused**	**Selective Treatment**	**Full Treatment**
	# of Respondents:	0	0	**118 (100%)**
	Advanced Airway			25 (21%)
	Bag Valve Mask			26 (22%)
	NIPPV			25 (21%)
	Supplemental oxygen			35 (30%)
	IV normal saline			49 (42%)
	IV Fentanyl			33 (28%)
	Naloxone			23 (19%)
	Contact Base Physician			30 (25%)
	Transport the patient to the hospital			115 (97%)
	Non-transport, Assess & Refer			3 (3%)
	Non-transport, Refusal of Service			3 (3%)
	Non-transport, AMA			5 (4%)
**Case #4:** A 72-year-old woman with advanced COPD feels short of breath and has increased work of breathing. Vital Signs P 125; RR, 32; SaO2, 79% on 4L; T, 37C; BP, 138/75. Her husband and health care surrogate states that she does not want continuous positive airway pressure (CPAP) but does want to go to the hospital **POLST Selection:** DNR/DNAR, Selective Treatment—Request transport to hospital only if comfort needs cannot be met in current location *Correct minimum:* Supplemental oxygen, Transport to the hospital		**Comfort Focused**	**Selective Treatment**	**Full Treatment**
	# of Respondents:	1 (1%)	**117 (99%)**	0
	Advanced Airway	0	0 (0%)	
	Bag Valve Mask	0	39 (33%)	
	NIPPV	0	9 (8%)	
	Supplemental oxygen	1 (1%)	106 (90%)	
	IV normal saline	0	49 (42%)	
	IV Fentanyl	0	21 (18%)	
	Naloxone	0	11 (9%)	
	Contact Base Physician	0	30 (25%)	
	Transport the patient to the hospital	1 (1%)	101 (86%)	
	Non-transport, Assess & Refer	0	3 (3%)	
	Non-transport, Refusal of Service	0	2 (2%)	
	Non-transport, AMA	0	7 (6%)	
**Scenario 5:** A 101-year-old man fell at home and was unable to get up independently or with the help of his wife, so she called 911 for assistance. Vital Signs P 85; RR 14; SaO2 96% RA; T 37C; BP 110/75. He has no physical complaints and does not want to go to the hospital **POLST Selection:** DNR/DNAR, Selective Treatment—Request transport to hospital only if comfort needs cannot be met in current location *Correct minimum:* Non-transport		**Comfort Focused**	**Selective Treatment**	**Full Treatment**
	# of Respondents:	1 (1%)	115 (97%)	2 (2%)
	Advanced Airway	0	0	0
	Bag Valve Mask	0	11 (9%)	1 (1%)
	NIPPV	0	7 (6%)	0
	Supplemental oxygen	0	15 (13%)	1
	IV normal saline	0	15 (13%)	1
	IV Fentanyl	0	13 (11%)	0
	Naloxone	0	8 (7%)	0
	Contact Base Physician	0	29 (25%)	0
	Transport the patient to the hospital	0	13 (11%)	1 (1%)
	Non-transport, Assess & Refer	1 (1%)	30 (25%)	0
	Non-transport, Refusal of Service	0	51 (43%)	1 (1%)
	Non-transport, AMA	0	51 (43%)	1 (1%)
**Scenario 6:** A 43-year-old male with pancreatic cancer on hospice feels very short of breath because of fluid that has accumulated in his lungs. Vital Signs P, 115; RR, 30; SaO2, 86% 2L home oxygen; T, 37 C; BP, 130/70. He states that he wants help with his symptoms, but does not want to go to the hospital **POLST Selection:** DNR/DNAR, Comfort focused *Correct minimum:* Non-transport		**Comfort Focused**	**Selective Treatment**	**Full Treatment**
	# of Respondents:	115 (97%)	2 (2%)	1 (1%)
	Advanced Airway	0	0	0
	Bag Valve Mask	10 (8%)	0	0
	NIPPV	27 (23%)	2	0
	Supplemental oxygen	93 (79%)	1	1
	IV normal saline	15 (13%)	0	0
	IV Fentanyl	24 (20%)	1 (1%)	0
	Naloxone	4 (3%)	0	0
	Contact Base Physician	68 (58%)	1	0
	Transport the patient to the hospital	8 (7%)	1 (1%)	1 (1%)
	Non-transport, Assess & Refer	19 (16%)	0	0
	Non-transport, Refusal of Service	16 (14%)	0	1 (1%)
	Non-transport, AMA	68 (58%)	1 (1%)	1 (1%)

*P*  pulse, *R* respiratory rate, *SaO2* oxygen saturation, *T* temperature, *BP* blood pressure, *IV*  Intravenous, *AMA* Against Medical Advice, *NIPPV*  Non-Invasive Positive Pressure Ventilation

When asked which “medical treatment/intervention is appropriate” for the patient based on the clinical information provided, advanced airways were correctly and appropriately avoided in patients in the vast majority of patients who selected *comfort focused treatment* or *selective Treatment* (Case #1,2, 4–6). For patients who selected *comfort focused treatment,* the majority of paramedics chose to administer supplemental oxygen (Case #2 (101;86%) #6 (93;79%) and a smaller minority chose to administer NIPPV (Case #2 (29;25%) #6 (27;23%). For Case #3, where a patient selected *full treatment*, but was overall clinically stable without hypoxia, paramedics selected invasive and NIPPV treatments in similar proportions (advanced airway (25;21%), bag valve mask (26;22%), NIPPV (25;21%), supplemental oxygen (35;30%)).

In terms of medication treatment, when a hospice patient who was short of breath chose *comfort focused treatment,* fentanyl was occasionally administered (Case #2 (49;42%), #6 (24;20%) and naloxone was infrequently but incorrectly administered (Case #2 (7;6%); #6(4;3%).

For all clinical scenarios, paramedics elected to use online medical oversight through base physician contact (13–68;11%-58%). The decision to contact the base physician tended to occur more often when there was an ultimate decision to not transport the patient. For example, in Case #2, the majority of respondents (73;62%) contacted base hospital and ultimately a minority (12; 10%) chose to transport the patient to the hospital. In contrast, in Case #1, a minority of respondents (27;23%) contacted base physician when the overwhelming majority (112;95%) ultimately decided on hospital transport.

In cases where the POLST indicated “*transport to hospital only if comfort needs cannot be met in current location*” (i.e. case #2) some paramedics chose to transport the patient anyway (12;10%) and the majority (68;58%) chose “Non-transport, Against Medical Advice (AMA)”. The minority of paramedics considered non-transport “Assess & Refer” (22;19%) or “Refusal of Service” (19;16%).

When paramedics self-assessed with a Likert scale confidence and confusion using the POLST, the majority of paramedics agreed that they knew how to use a POLST to “decide which medical interventions to provide” (106;90%) and “to determine whether to transport a patient” (74;67%). However, after completing the cases, similar proportion of paramedics agreed (42;36%), disagreed (43;36%), or were neutral (30;25%) when asked if they find the POLST confusing. ANOVA tests comparing mean years of EMS experience across the Likert responses were not significantly (p < 0.05) associated with self-assessed confidence or confusion in using the POLST.

### Limitations

A major limitation of study design is that these are fictional clinical cases and its extrapolation to real world scenarios is limited. In real clinical scenarios there would be more information available to make treatment and transportation decisions (i.e. ability to talk to patients/family about preferences). However, these findings do corroborate conclusions of a systematic review in which there was a moderate strength of evidence to support treatment limitations on POLST may reduce treatment intensity among patients with serious illness [8].

Another major study limitation is potential participation bias, particularly given small overall sample size. Survey respondents might have predisposing training experiences or interest in the topic that might limit generalizability. Moreover, since the methodology used a convenience sample, it is unknown how many total EMS providers received the survey and the overall response rate. The small sample size has the potential to result in underpowering.

A limitation of the study design is that it allowed respondents to select multiple answers even if discordant. Although this design was intended to allow respondents to maximize treatment and transportation options it does limit analysis and obscures what a participants first action might be. For example, if a paramedic used their contact with a base physician to inform decisions about transporting, the survey was unable to capture this decision-making sequence. Another potential survey design flaw, not discovered during piloting, is task misinterpretation. For example, in a hemodynamically stable patient that wanted *full treatment* (Case #3), many paramedics (25;21%) chose an advanced airway. This is clinically incorrect in the scenario and also unlikely that paramedics would intubate in reality. Perhaps, respondents misinterpreted the task and intended that an advanced airway would be appropriate if the patient decompensated.

Additionally, since both EMS systems and POLST have significant geographic variation, it is unclear how generalizable these findings are beyond California. For example, many states POLST forms do not have a dedicated sub-selection box that states “*transport to hospital only if comfort needs cannot be met in current location*”, although the National POLST does integrate similar language [2].

## Discussion

Paramedics are confident in their ability to use a POLST to decide appropriate medical interventions and make transportation decisions. However, after completing the cases, many paramedics felt the POLST confusing. If only 47 (40%) of paramedics reported prior formal POLST training, this demonstrates a major curriculum gap and an area for targeted future trainings. The findings of this study are overall consistent with previous literature that demonstrates a paucity of formal training and guidelines around caring for patients near the end of life [10, 11, 12].

When the California POLST states “*transport to hospital only if comfort needs cannot be met in current location*”, this requires a nuanced and complex clinical assessment. Paramedics must not only assess if a patient’s comfort needs are being met, but decide if transportation to the hospital might provide that comfort. If a hospice patient (Case #2 and #6) is having difficulty with symptom management, transportation to the hospital might not be in the best overall interest of the patient. While fentanyl should be liberally administered to hospice patients in pain or respiratory distress, naloxone and non-invasive positive pressure should not be routinely considered. The development of specific hospice or palliative medicine EMS protocols can reinforce appropriate treatment pathways for these patients that emphasize comfort treatment [13]. In order for paramedics to effectively use the POLST for transportation decisions, paramedic POLST education and training should emphasize communication skills and basic end of life symptom management. For hospice patients, the intention might be to die at home, however fear, grief, distressing symptoms during the dying process, slow hospice response times may cause families to call the number they are familiar with, 9–1-1 [14]. Communication skills to confirm and clarify goals of care during acute crisis, in particular transportation, is well within the scope of practice of paramedics, but this skill benefits from communication training. Similar communication skills training has been successful for death notification [15], and can even have a protective effect for EMS clinicians against burnout [16]. When empowered with the communication tools, interactions can be rewarding rather than morally distressing [13].

For patients with serious illness, for whom hospital transport is not within their treatment goals, these patients should not be designated “AMA”. Notably, there is local variation in protocols for non-transported patients and not all local protocols allow for “Assess and Refer.” In cases where local protocol permits and hospice is available for referral, “Assess and Refer” might be a more appropriate designation. The term “AMA” is not appropriate for scenarios near the end of life, since their decision for non-transport is a reasonable medically indicated alternative. Improving EMS care for patients with serious illness involves a paradigm cultural shift that can begin by protocolizing simple language changes. Particularly for hospice patients, specific EMS guidelines that emphasize comfort and “Assess and Refer” to hospice might be helpful (Appendix2). Certainly, such a cultural change must also be reinforced by a financial reimbursement system that values non-transport of these select patients and recognizes paramedics for nuanced end of life care they might be providing.

Many paramedics chose to contact the base hospital physician, which suggests they found the scenarios ethically, legally or clinically challenging which is consistent with local guidelines. In general, paramedics tended to call the base hospital physician more when not transporting a patient. This is similar to the use of online medical oversight for informed refusals [17–19]. Overall, this underscores the necessity that base hospital physicians should be well versed in the POLST, as well as with end of life care symptom management and goals of care communication in order to provide paramedics support.

In conclusion, the POLST is a powerful tool for paramedics when caring patients with serious illness. Although paramedics are confident in their ability to use a POLST to decide appropriate medical interventions, many still find the POLST confusing particularly when making transportation decisions. The authors recommend further research of EMS POLST utilization and goal concordant care, dedicated paramedic POLST education, specific EMS hospice and palliative care protocols, and better nomenclature for non-transport in order to improve care for patients with serious illness and their families.

## Supplementary Information

**Additional file 1. **

**Additional file 2.**

## Data Availability

Raw data available as supplement.

## Abbreviations

POLST: Physician Order for Life-Sustaining Treatment
EMS: Emergency Medical Services
ANOVA: Analysis of variance
NIPPV: Non-Invasive Positive Pressure Ventilation
AMA: Against Medical Advice
P: Pulse
R: Respiratory rate
SaO2: Oxygen saturation
T: Temperature
BP: Blood pressure
IV: Intravenous
