# Supplementary material for: Paramedic use of the Physician Order for Life-Sustaining Treatment (POLST) for medical intervention and transportation decisions
Source: BMC Emerg Med. 2022 Aug 11;22:145. doi: 10.1186/s12873-022-00697-3 (PMC9367154; doi:10.1186/s12873-022-00697-3)
Supplement: Supplementary file 2 — Additional file 2. [file 12873_2022_697_MOESM2_ESM.docx]

We are asking you to take part in a research study conducted by [PI *Amelia Breyre*] at the University of California, San Francisco. Participating in this study is optional.

If you choose to be in the study, you will complete a 15-minute survey about your perspective on caring for patients with a serious illness.

We will keep your answers confidential and will not share your personal information with anyone outside the research team.

The first 500 submissions will receive a $20 emailed amazon gift card.

Questions? Please contact Amelia Breyre at [amelia.breyre@ucsf.edu](mailto:amelia.breyre@ucsf.edu). If you have questions or concerns about your rights as a research participant, you can call the UCSF Institutional Review Board at 415-476-1814.

If you want to participate in this study, click the [*Agree, Accept, Next, Start*] button to start the survey.

1. **Statements**

**Please rate the following statements:**

(Likert: Strongly disagree, disagree, neutral, agree, strongly agree)

1. **I know how to use a POLST to determine whether to resuscitate a patient**
2. **I know how to use a POLST to determine which medical interventions to provide**
3. **I know how to use a POLST to determine whether to transport a patient**
4. **SCENARIOS – PART B (Medical Intervention) - PARAMEDIC**

For each case, read the associated POLST and clinical scenario. Select what type of medical intervention is selected. Then determine which treatment/intervention is appropriate for the patients stated preference.

**After review of the patient POLST, what type of medical interventions is selected:**

- - Full Treatment
  - Selective Treatment
  - Comfort Focused Treatment

Based on the information provided, which of the following is appropriate treatment/intervention for this patient. Check all that may apply:

- Advanced airway (i.e Intubation/ Supraglottic airway placement (i.e. IGel)
- Bag Valve Mask (BVM)
- Non-Invasive Positive Pressure
- Supplemental Oxygen
- IV Fentanyl
- IV Normal Saline
- Naloxone
- Transport the patient to the hospital
- Non-transport, Against Medical Advice
- Non-transport, Assess & Refer
- Non-transport, Refusal of Service
- None of the above

| **Case #1:**  A 95-year-old female with dementia lives with her family is more confused than her baseline. She has foul smelling diapers and a chronic pressure sacral ulcer. Vital Signs P, 105; RR 12; SaO2, 97%; T, 39 C; BP, 90/50. The family states that they would like her to be evaluated for antibiotics, but do not want admission to the hospital.  POLST: DNR/DNAR, Selective Intervention | **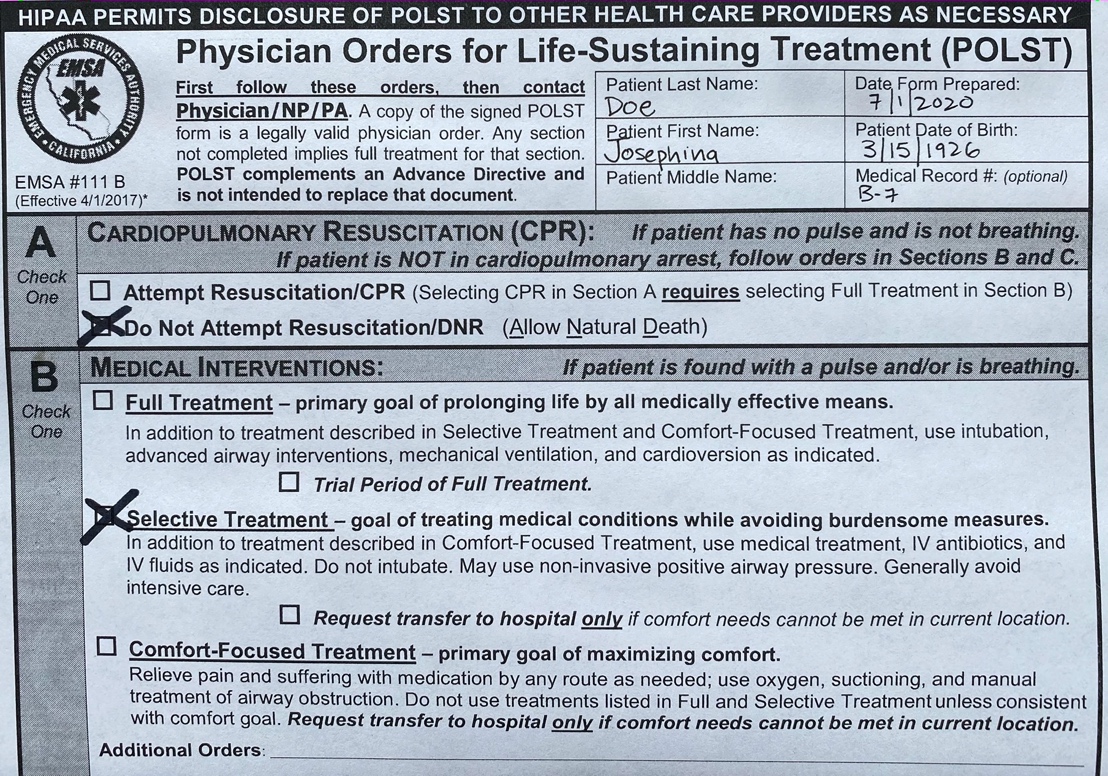** |
| --- | --- |
| **Case #2:**  A 43-year-old female with metastatic ovarian cancer on hospice feels very short of breath because of fluid that has accumulated in her lungs. Vital Signs P, 115; RR, 30; SaO2, 86% 2L home oxygen; T, 37 C; BP, 130/70. She states that she wants help with her symptoms, but does not want to go to the hospital.  POLST: DNR/DNAR, Comfort focused treatment | **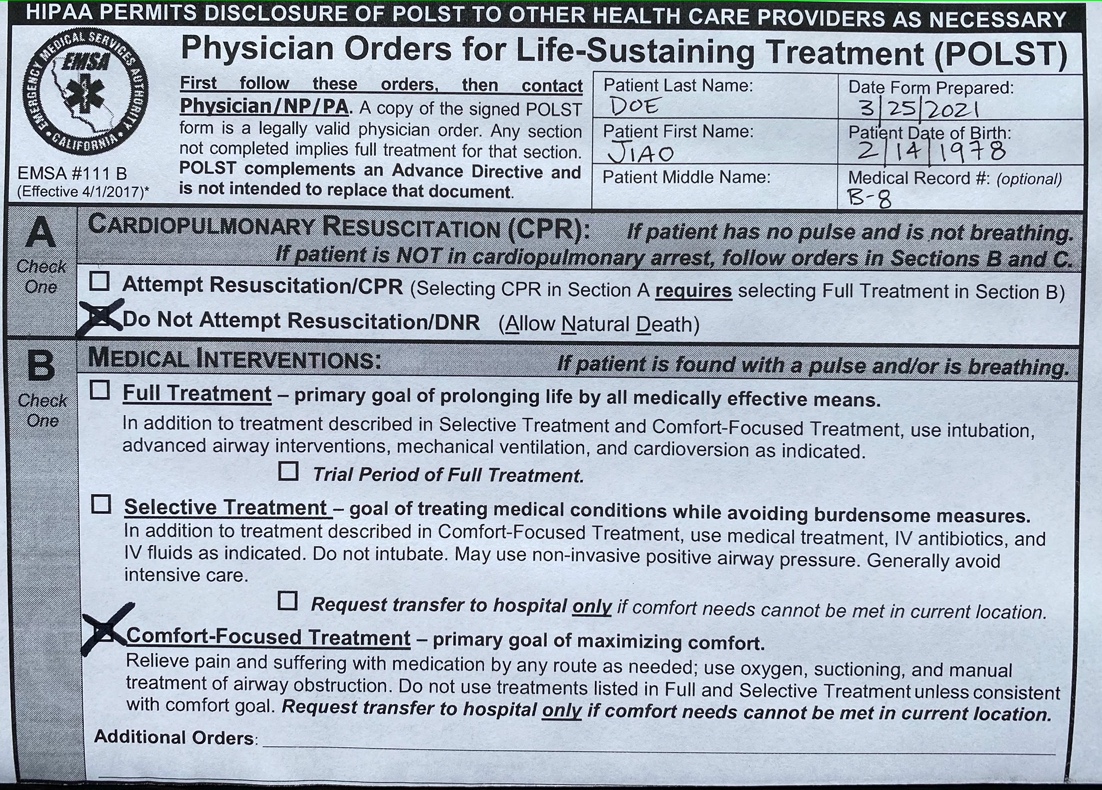** |
| **Case #3:**  A 87-year-old male with advanced dementia, on apixaban for atrial fibrillation hits his head at his nursing facility and now has a scalp hematoma. He is awake and talking and to staff at his mental status baseline. Vital Signs P, 75; RR, 12; SaO2, 99%; T, 37 C; BP, 115/75.  POLST: DNR/DNAR, Full treatment | **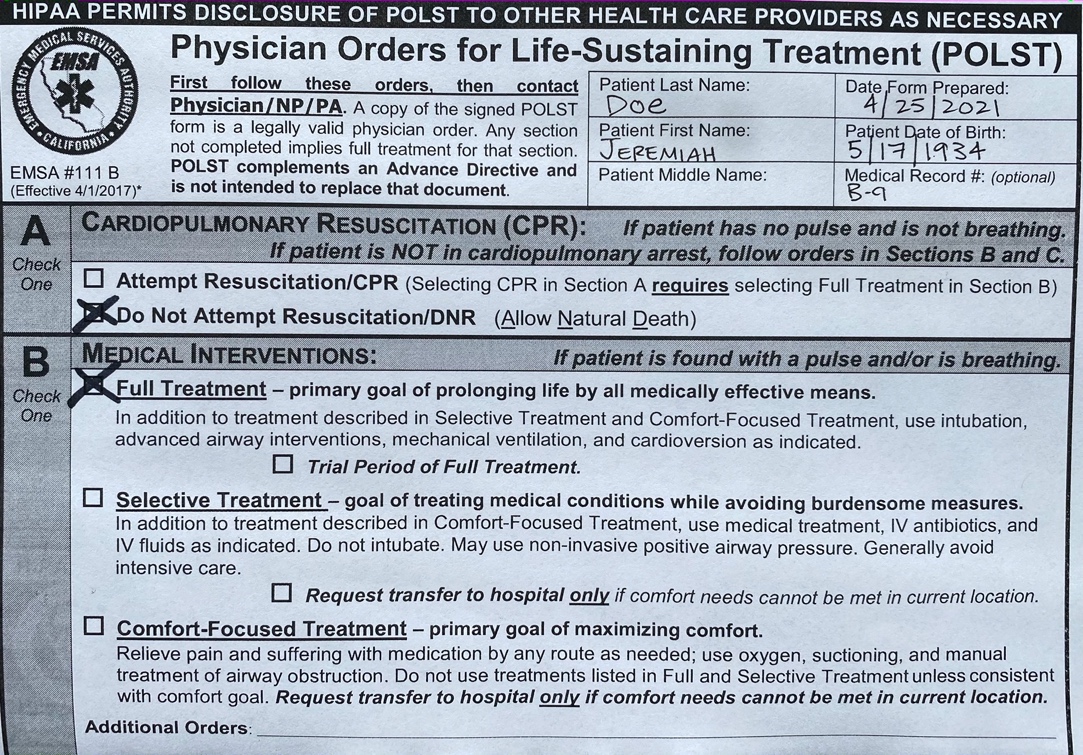** |
| **Case #4**  A 72-year-old woman with advanced COPD feels short of breath and has increased work of breathing. Vital Signs P 125; RR, 32; SaO2, 79% on 4L; T, 37C; BP, 138/75. Her husband and health care surrogate states that she does not want positive airway pressure but does want to go to the hospital.  DNR/DNAR, Selective Intervention, request transfer to hospital only if comfort is not met at home | **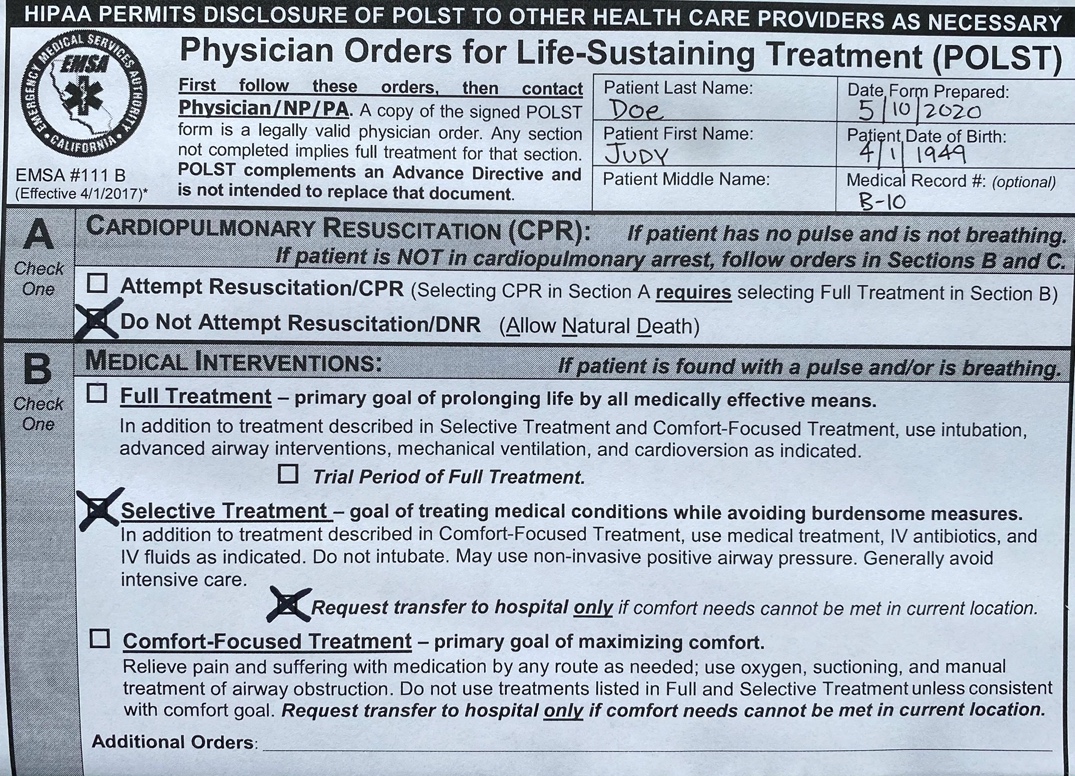** |
| **Case #5:**  A 101-year-old man fell at home and was unable to get up independently or with the help of his wife, so she called 911 for assistance. Vital Signs P 85; RR 14; SaO2 96% RA; T 37C; BP 110/75. He has no physical complaints and does not want to go to the hospital.  DNR/DNAR, Selective Intervention, request transfer to hospital only if comfort is not met at home | 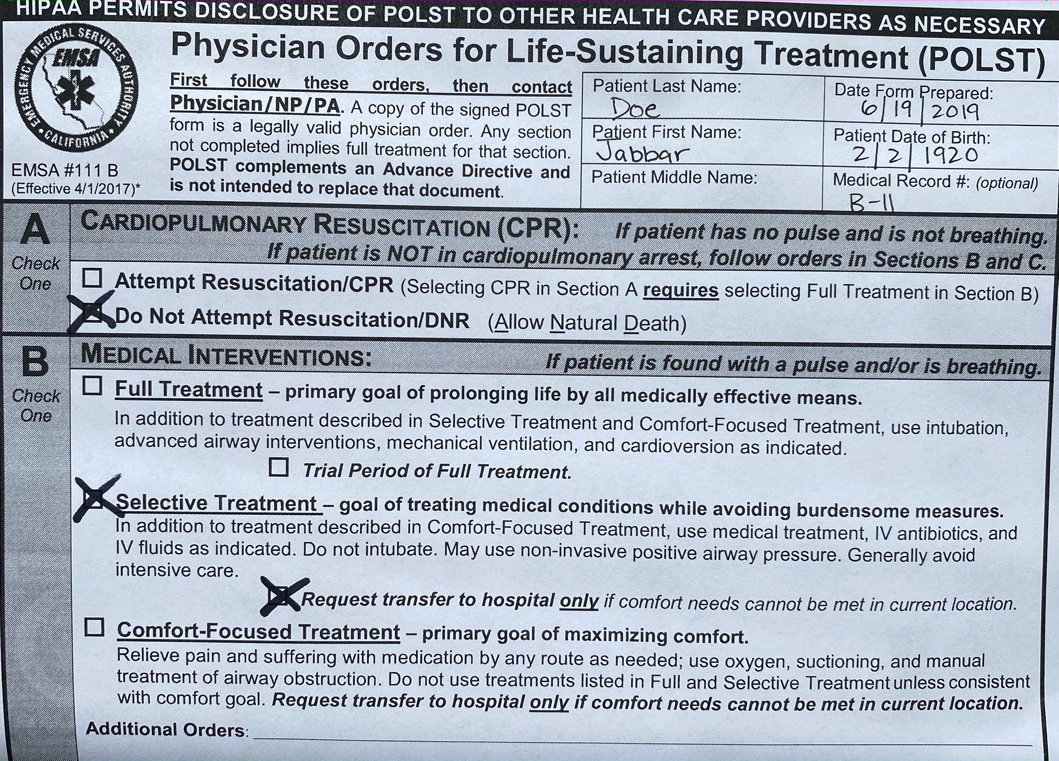 |
| **Case #6**  A 43-year-old male with pancreatic cancer on hospice feels very short of breath because of fluid that has accumulated in his lungs. Vital Signs P, 115; RR, 30; SaO2, 86% 2L home oxygen; T, 37 C; BP, 130/70. His states that she wants help with his symptoms, but does not want to go to the hospital.  POLST: DNR/DNAR, Comfort focused treatment | **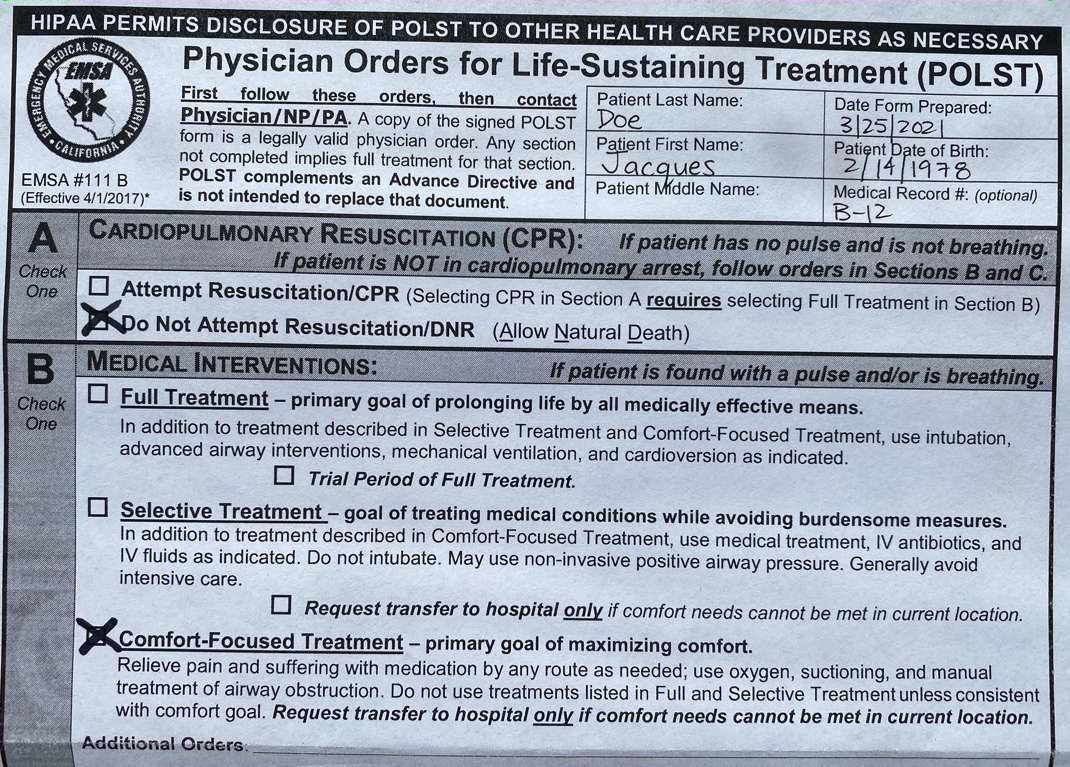** |

**Part III:** *Please answer the following questions based on the most recent patient you treated with a POLST form*:

- 1. **Where was the patient located at the time you responded to the call?**

Private home, Residential Care/Assisted Living Facility, Foster home, nursing facility, other

- 1. **Where was the patient’s POLST form located?**

Front of chart, refrigerator, wallet card in wallet, provided by family member, other (please specify), unable to find

- 1. **Was this patient’s POLST form filled out appropriately?** Yes, No – signature missing, no-conflicting orders, No section A and B not marked, other
  2. **Did the POLST form change the treatment plan from treatments that would otherwise have been given to this patient?** Yes, no, unsure, other
  3. **Was this patient’s POLST form over-ridden?** No, Yes- by patient, yes-by family member/ durable power of attorney over healthcare, Yes- nursing facility staff, other
  4. **Was this case typical of situations where POLST form is present?** No, yes
  5. **Please rate how you agree with the following statement: “I find POLSTs confusing”:** Strongly disagree, disagree, neutral, agree, strongly agree

**Part IV: DEMOGRAPHICS**

1. **Age:** short answer text
2. **Gender**: female, male, non-binary
3. **Local EMSA/County**: Alameda, San Francisco, San Mateo, Contra Costa, Costal Valleys, Santa Clara
4. **Agency**: Falck, Alameda County Fire Department, Alameda City Fire Department, Berkeley Fire Department, Oakland Fire Department, Hayward Fire Department, Freemont Fire Department, San Francisco Fire Department, AMR-SF, AMR-San Mateo, AMR-Contra Costa, King American, Contra Costa Fire Department, Sonoma Fire Department, Other (please specify)
5. **Level of training**: EMT, Paramedic
6. **#Years working in EMS:**
7. **Have you had prior training on POLST or other related palliative training**? No, yes-self training, yes- formal didactics, other
8. **What would be the ideal method for additional training on this topic?** Video, online module, simulation training with actors, hybrid, other

Email address (This is where an amazon gift card will be sent to within the next 1 weeks)
